# Translabial ultrasound: a non-invasive technique for assessing “technical errors” after TOT failure

**DOI:** 10.1007/s00192-021-04897-6

**Published:** 2021-06-30

**Authors:** Ester Illiano, Francesco Trama, Vincenzo Li Marzi, Vito Mancini, Giuseppe Carrieri, Claudia Collà Ruvolo, Gianluigi Califano, Consuelo Fabi, Stefano Brancorsini, Elisabetta Costantini

**Affiliations:** 1 Department of Surgical and Biomedical Science, Andrological and Urogynecological Clinic, Santa Maria Terni Hospital, University of Perugia, Viale Tristano di Joannuccio, 05100 City Terni, TR Italy; 2 Urology Clinic, Careggi Hospital, University of Florence, Florence, Italy; 3 Department of Urology and Renal Transplantation, University of Foggia, Foggia, Italy; 4 Department of Neurosciences, Reproductive Sciences and Odontostomatology, University of Naples Federico II, Naples, Italy; 5 Department of Experimental Medicine, Section of Terni, University of Perugia, Perugia, Italy

**Keywords:** Translabial ultrasound, TOT, Stress urinary incontinence, Bladder neck funneling, Concordance of urethral movement

## Abstract

**Introduction and hypothesis:**

The aims of this study were to evaluate by transperineal ultrasound if there were ultrasound-detectable changes over time in the dynamic behavior of the sling in patients who underwent transobturator tape (TOT), and to evaluate if dynamic translabial ultrasonography recognized factors that may be associated with failed surgery.

**Methods:**

This was a single-center prospective study. We included women who underwent “out-in” TOT for stress urinary incontinence (SUI). A dynamic translabial ultrasound was performed 6 months post-surgery and again at the last visit. The objective cure for SUI was defined as the absence of urine leakage during the stress test. We evaluated the bladder neck mobility at rest and during Valsalva; the position of the mesh along the urethra; the concordance of urethral movement with the sling during Valsalva; the symmetry of the lateral arms of the sling during straining; and the presence or absence of bladder neck funneling.

**Results:**

From December 2012 to February 2016, 80 consecutive patients were included. Six months after surgery, incontinent women compared with continent women had the sling in a proximal or distal position, that moved discordantly with the urethra (*p* < 0.0001), with asymmetry arm and bladder neck funneling (*p* < 0.0001). Continent patients had a significant improvement of urethrocele grade both at rest (*p* = 0.036) and during Valsalva (*p* = 0.045).

**Conclusions:**

Technical and positioning errors can lead to the failure of anti-incontinence surgical treatment. Translabial ultrasound allows the correct positioning of the sling to be evaluated and any errors that need to be analyzed in order to then solve the failure.

## Introduction

The pelvic floor muscles contribute to urinary continence by providing external support to the urethra [1, 2]. When abdominal pressure increases owing to a cough, sneeze, or other physical stress, the pelvic floor muscles contract. Contraction of these muscles results in mid-urethral closure as the pubococcygeus muscle stretches the distal vagina and the proximal urethra stretches around a competent pubourethral ligament [3]. Failure of this urethral closure mechanism may contribute to the pathophysiology of stress urinary incontinence (SUI) [4].

Ulmsten and Petros introduced the mid-urethral sling (MUS) as a surgical replacement for a defective pubourethral ligament [5, 6]. Despite good long-term outcomes of MUS procedures [7], 5–23% of patients who receive an MUS have persistent or recurrent urinary incontinence [8]. Several risk factors for persistent incontinence after MUS implantation have been investigated, including age, obesity, previous anti-incontinence surgery, and concomitant prolapse surgery [8]. However, the position of the sling along the urethra, as well as the dynamic interaction between the sling and urethra, seem to be crucial for obtaining a successful surgical outcome.

Many studies have used two-dimensional transperineal or translabial ultrasound to explore the location and tension of the sling and ensure continence after implanting tension-free vaginal tape (TVT) slings [9–11]. A few studies have evaluated the dynamic behavior of mid-urethral transobturator tape (TOT) slings and have characterized how the sling and urethra respond to increased intra-abdominal pressure [12]. However, these studies have not explored the long-term ultrasound characteristics of TOT slings.

The primary outcome of this study was to evaluate, via dynamic translabial ultrasonography, if there were any long-term, ultrasound-detectable changes in the dynamic behavior of the sling in patients who underwent TOT.

The secondary outcome was to evaluate if dynamic translabial ultrasonography could be used to recognize improper positioning or dislodgment of the tape or other factors that may be associated with failed surgery.

## Materials and methods

This was a single-center prospective study. The local ethics committee (IRB) approved the study (CEAS UMBRIA N:2564/15) and all patients signed an informed consent document. We included women who underwent “out-in” TOT for SUI. Women with a history of radical pelvic surgery or previous pelvic organ prolapse (POP) surgery, or with POP stage ≥II, were excluded. The preoperative evaluation included a medical history, a clinical examination using the POP-Q classification [13], a standardized cough stress test (CST) performed in the standing position at a bladder volume of 300 ml, a urodynamic study according to International Continence Society (ICS) criteria, and a dynamic translabial ultrasound. We performed the urodynamic test on all patients because it may provide important information for the surgeon and the patient. SUI was defined according to ICS standards and classified according to the Ingelman-Sundberg scale [14].

Transobturator tape surgery was performed by a senior surgeon (EC) following the technique originally described by Delorme [15]. Patients were seen for follow-up visits 1, 3, 6, and 12 months after surgery and annually thereafter, with the most recent (“last”) visits in June or July 2020, by an independent urologist (FT). He was different urologist from the surgeon and the urologist who performed the ultrasound. Each follow-up visit included a medical history, physical examination, and uroflowmetry with PVR measurement. A dynamic translabial ultrasound was performed 6 months post-surgery and again at the last visit. Urinary symptoms were evaluated using the standardized Urogenital Distress Inventory Short Form Questionnaire (UDI-6) [16]. Voiding symptoms were evaluated using question five of UDI-6, where patients report their difficulty voiding on a four-point scale, as well as with additional yes/no questions about hesitancy, strength of urine stream, intermittency, straining to void, and feeling of incomplete bladder emptying. Storage symptoms were identified in accordance with the International Urogynecological Association (IUGA)/ICS [17].

The objective cure for SUI was defined as the absence of urine leakage during the stress test.

### Dynamic translabial ultrasound

Ultrasound was performed with the patient at rest in the dorsal lithotomy position, with the hips flexed and abducted and the bladder comfortably full. The procedure was then repeated during a maximum Valsalva maneuver. We used a 3.5–5 MHz curved array probe that we covered with a glove and then coated with water-soluble gel. The probe was placed between the labia. All ultrasounds were performed by an independent urologist (EI).

**Fig. 1:**
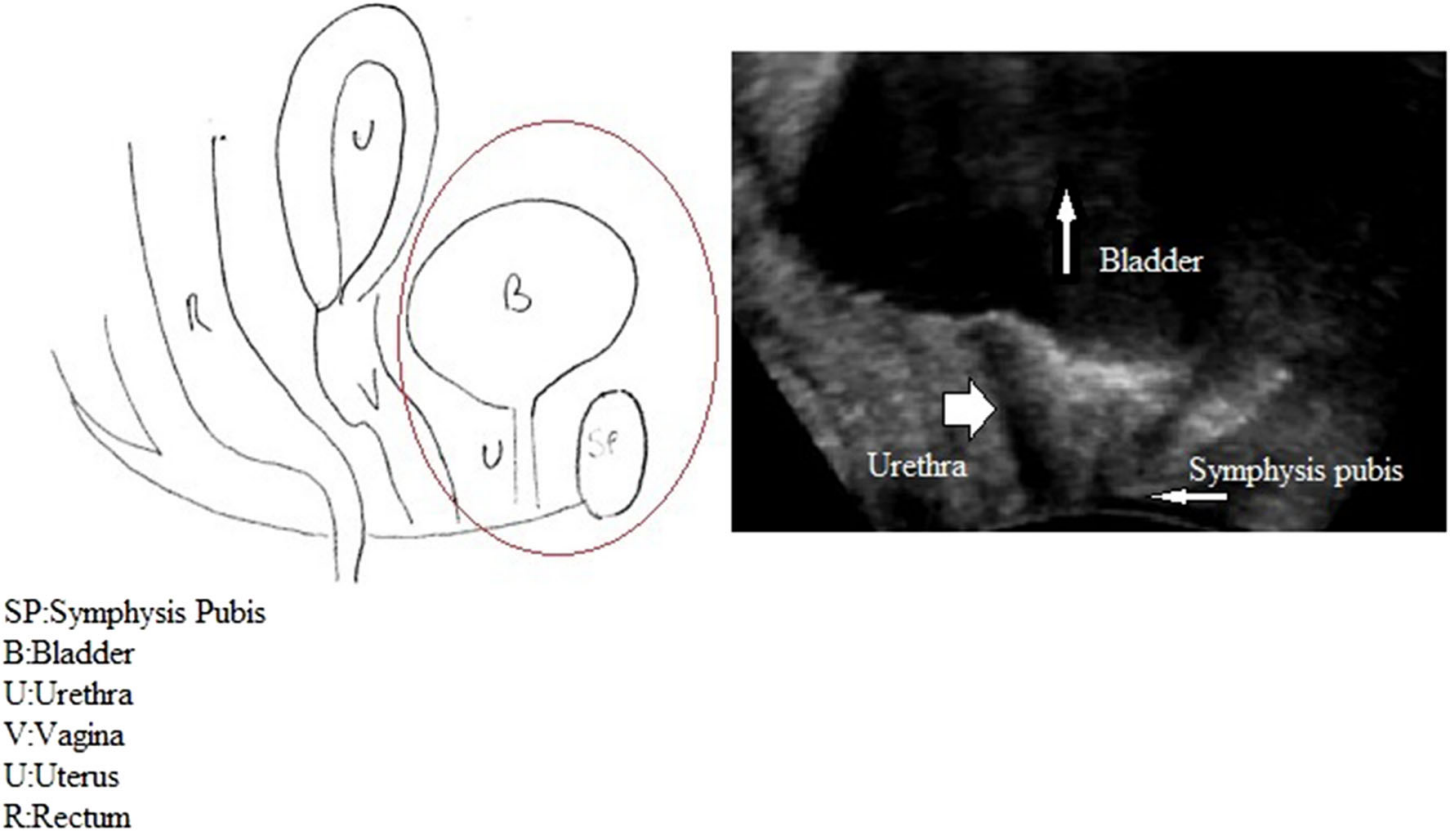
Pelvic structures on transperineal ultrasound

The ultrasound images showed the symphysis pubis, urethra, bladder, vagina, and rectum (Fig. 1). The symphysis pubis, shown anteriorly on the right in Fig. 1, was used as a landmark to evaluate bladder neck position and mobility. We preferred to orient images with cranial structures at the top.

**Fig. 2:**
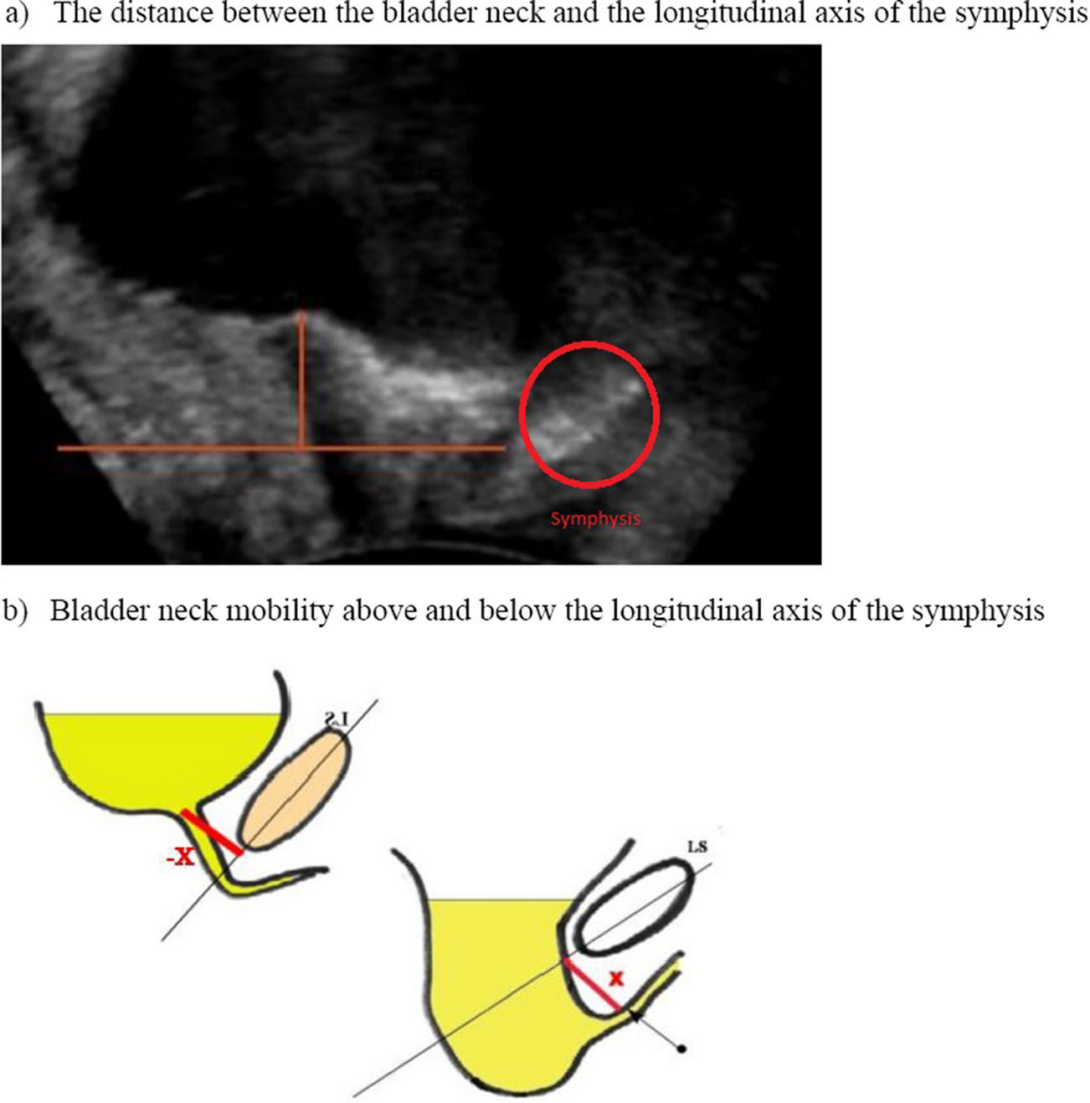
Bladder neck motility. **a** The distance between the bladder neck and the longitudinal axis of the symphysis. **b** Bladder neck motility above and below the longitudinal axis of the symphysis

To assess bladder neck mobility, we measured the distance between the bladder neck and the longitudinal axis of the symphysis (Fig. 2a). We recorded distances above and below the longitudinal axis of the symphysis as negative and positive respectively (Fig. 2b). Bladder neck mobility was measured both at rest and during Valsalva. The difference in bladder neck mobility between rest and Valsalva was also measured to assess bladder neck descent.

Additional parameters evaluated at post-operative ultrasound were:

1. The position of the mesh along the urethra
2. The concordance of urethral movement with the sling during Valsalva
3. The symmetry of the lateral arms of the sling during straining
4. The presence or absence of bladder neck funneling (i.e., open bladder neck on Valsalva or coughing)

The position of the sling along the urethra was determined relative to the urethral length. A sling was considered “proximal” if it was located along the first 0–40% of the urethra, measured from the bladder neck. Slings were “mid-urethral” if they were located along the second 40–60% of the urethra, and “distal” if they were located along the final 60–100%. The sling was identified as a hyperechoic sub-urethral image. Urethral length was measured from the urethrovesical junction to the external urethral meatus in the mid-sagittal plane. Because the urethra forms an angle, we calculated the total urethral length by summing the lengths of the straight portions.

The concordance of urethral movement with the sling during Valsalva was evaluated by comparing the location of the sling at rest, measured relative to the urethral length, to the location of the sling at maximum Valsalva. Urethral movement was considered concordant with the sling if the sling location at maximum Valsalva was identical to the sling location at rest. If not, urethral movement was considered discordant with the sling.

For statistical analysis, we assessed post-operative incontinence by categorizing patients as either “dry” or “wet.” We considered patients “wet” if they had any kind or grade of leakage.

### Statistical analysis

Statistical analysis was performed using SPSS v.23 and Med-Calv v.18. A logistic regression model and odds ratios (with 95% confidence intervals) were used to assess the possible predictive factors for treatment failure. To determine the statistical significance of categorical data comparisons, we performed the Chi-squared test with a continuity correction for each 2 × 2 contingency table. Fisher’s exact test was used when expected frequencies were insufficient for a Chi-squared test. We considered *p* < 0.05 to be statistically significant.

## Results

From December 2012 to February 2016, a total of 110 consecutive patients underwent TOT. Thirty of these patients were lost to follow-up, leaving 80 patients who were assessed at the last follow-up in 2020. Table 1 shows the clinical and demographic characteristics of this study population. The last follow-up visit was, on average, 72 months after TOT (range: 54–92 months). The objective cure rate was 80% (64 patients) 6 months after surgery and 77.5% (62 patients) at the last visit. The subject cure rate was 76% (61 patients). They answered “not at all” to item 3 of UDI-6.

**Table 1:** Clinical and demographic characteristics of this study population

Characteristics	*N* = 80
Age (mean ± SD) years	52 ± 11.5
Body mass index, kg/m^2^	23.5 ± 1.2
Parity median (range)	3 (1–5)
Menopause, *n* (%)	60 (75)
Smoking, *n* (%)	25 (31.5)
Concomitant surgery for POP, *n* (%)	0
Previous anti incontinence surgery, *n* (%)	0
Voiding symptoms, *n* (%)	0
Storage symptoms, *n* (%)	23 (28.7)
Urgency urinary incontinence, *n* (%)	45 (56.2)

Tables 2 and 3 show the postoperative ultrasound parameters for continent and incontinent patients evaluated 6 months after TOT surgery and at their last visit respectively. Six months after surgery, continent women had a significantly improved urethrocele grade, both at rest (*p* = 0.036) and during Valsalva (*p* = 0.045) compared with incontinent women. Continent women also showed less urethral movement (bladder neck descent) from rest to maximum Valsalva (*p* = 0.046).

**Table 2:** Postoperative ultrasound parameters evaluated in incontinent and continent women 6 months after TOT surgery

Ultrasound parameters	Continent women			Incontinent women		
	Baseline	Post surgery	*p* value	Baseline	Post surgery	*p* value
UR	−3.2 ± 42.3	−14.6 ± 9.5	0.036	−6.3 ± 15.3	−12.7 ± 11.8	0.23
UV	−7.3 ± 5.6	−9 ± 5.1	0.045	−8.1 ± 3.9	−10.8 ± 6.8	0.26
UV − UR	10.3 ± 11.5	6.7 ± 10.5	0.046	8.4 ± 6.6	5.0 ± 15.3	0.43

*UR* urethrocele at rest, *UV* urethrocele during maximum Valsalva maneuver

**Table 3:** Postoperative ultrasound parameters evaluated in incontinent and continent women at their last visit

Ultrasound parameters	Continent women			Incontinent women		
	Post surgery	Last visit	*p* value	Post surgery	Last visit	*p* value
Urethrocele at rest (UR)	−14.6 ± 9.5	−13.7 ± 7.5	0.9	−12.7 ± 11.8	−11.2 ± 9.3	0.9
Urethrocele during maximum Valsalva maneuver (UV)	−9 ± 5.1	−8.3 ± 4.2	0.9	10.8 ± 6.8	−9.3 ± 6.2	0.9
UV − UR	6.7 ± 10.5	7.3 ± 9.4	0.9	5.0 ± 15.3	6.2 ± 14.1	0.9

Bladder neck mobility, both at rest and at Valsalva, increased slightly in the 6 years between the immediate and long-term postoperative visits; however, this increase was not statistically significant (*p* = 0.9; Table 3). Bladder neck descent also showed a nonsignificant increase between the immediate and long-term post-operative visits (6.7 ± 10.5 versus 6.3 ± 10.1). These increases in bladder neck mobility and descent were not associated with clinical deterioration, as patients were still continent.

Incontinent women had the sling in a more proximal (31.3% vs 1.6%, *p* = 0.001) or distal (68.8% vs 3.1%, *p* < 0.0001) position than continent women (Table 4). The position of the sling did not change over time.

**Table 4:** Characteristics of the sling by transperineal ultrasound in continent and incontinent women

Ultrasound parameters	Continent women	Incontinent women	Odds ratio (95% CI)	*p* value
Post-operative open bladder neck	9 (14.1)	7 (43.8)	4.75 (1.41–15.99)	0.014
Asymmetry of sling arm	2 (3.1)	12 (75.0)	93 (15.27–566.24)	<0.0001
Mesh position				
Proximal	1 (1.6)	5 (31.3)	28.63 (3.04–269.13)	0.001
Medium	61 (95.3)	0	0.04 (0.01–0.14)	<0.0001
Distal	2 (3.1)	11 (68.8)	68.20 (11.72–396.70)	<0.0001
Concordance of urethral movement with sling on Valsalva				
Concordant	64 (100)	1 (6.3)	16.00 (2.39–106.73)	<0.0001
Discordant	0	5 (31.3)	1.45 (1.04–2.02)	<0.0001
UVJ moves distal to the sling	0	10 (62.5)	2.66 (1.41–5.02)	<0.0001
Bladder neck funneling	3 (4.7)	8 (55)	20.33 (4.45–92.78)	<0.0001

*UVJ* urethrovesical junction

The sling moved concordantly with the urethra in all continent patients compared with only one incontinent patient (100% vs 6.3%, *p* < 0.0001), both 6 months and 6 years after surgery (Table 4). In 10 incontinent patients, the urethrovesical junction moved distal to the sling at Valsalva. In five incontinent patients, the urethra moved in a discordant manner from the sling; in these women the sling was located beneath the proximal urethra. These characteristics of the sling did not change over time.

Sling arm asymmetry and post-operative bladder neck funneling were both more prevalent in incontinent women than in continent women (*p* < 0.0001 for both comparisons). Women with an open bladder neck had more distal slings than women with a closed bladder neck (36.4% vs 0%, *p* < 0.0001).

Two women became incontinent with stress during follow-up. These patients had a serious intrinsic sphincter deficiency at the urodynamics test; their slings were located beneath the distal urethra and moved concordantly with the urethra.

At the last visit, 8 continent women (12.5%) had de novo voiding symptoms. In all these patients, the sling was located beneath the proximal urethra. No patients exhibited a PVR >50 ml or a urodynamic obstruction according to the Blaivas–Groutz nomogram [18].

Storage symptoms persisted in 6 continent women (9.4%, *p* < 0.0001), and the sling was located at the proximal urethra in only one of these patients.

The urgency of urinary incontinence persisted in 20 patients (44.4%, *p* < 0.0001), and 2 patients had de novo episodes. All these patients had their sling located beneath the mid-urethra.

We used univariate and multivariate logistic regression analysis to assess whether different characteristics of the sling were risk factors for the failure of the anti-incontinence surgery. Results from these analyses are listed in Table 5. These analyses showed that asymmetric sling arms, a distal and proximal sling position, the distal movement of the urethrovesical junction and the discordant urethral movement from the sling were risk factors for the failure of TOT. The bladder neck funneling has not been confirmed by the multivariate analysis as a risk factor.

**Table 5:** Univariate analysis and multivariate logistic regression final model for failure of the anti-incontinence surgery vs ultrasound parameters

Ultrasound parameters	Univariate analysis		Multivariate analysis	
	Odds ratio (95% CI)	*p* value	Odds ratio (95% CI)	*p* value
Post-operative open bladder neck	4.75 (1.41–15.99)	0.014	0.60 (0.03–0.07)	0.54
Asymmetry of sling arm	93 (15.27–566.24)	<0.0001	4.47 (0.10–0.27)	<0.0001
Mesh position				
Proximal	28.63 (3.04–269.13)	0.001	4.71 (0.15–0.38)	<0.0001
Medium	0.04 (0.01–0.14)	<0.0001		
Distal	68.20 (11.72–396.70)	<0.0001	2.31 (0.01–0.22)	0.02
Concordance of urethral movement with sling on Valsalva				
Concordant	16.00 (2.39–106.73)	<0.0001		
Discordant	1.45 (1.04–2.02)	<0.0001	12.78 (0.71–0.97)	<0.0001
UVJ moves distal to the sling	2.66 (1.41–5.02)	<0.0001	11.76 (0.56–0.79)	<0.0001
Bladder neck funneling	20.33 (4.45–92.78)	<0.0001	1.74 (0.13–0.009)	0.08

*UVJ* urethrovesical junction

## Discussion

This study showed that sling position, bladder neck mobility and funneling, and the concordance of urethral movement with the sling did not change over time, even 6 years after the implantation of the sling. A TOT sling with symmetrical arms that is implanted under the middle of the urethra, and that moves concordantly with the urethra, is most likely associated with good long-term outcomes. There are no prospective studies in the literature that evaluate the long-term ultrasound features after a TOT implant or their correlations with functional outcomes. Most studies have been performed on patients undergoing TVT [17–19] and have only a short or moderate follow-up period [20].

Hegde et al. performed a case–control study on women who underwent TOT and evaluated the ultrasound features at a 1-year follow-up visit [11]. Our long-term results agree with the 1-year outcomes reported by Hegde et al. They showed that, 1 year after TOT, continent women had the sling positioned along the middle of the urethra more frequently than patients with persistent incontinence (*p* < 0.0001) [11]. These observations disagree with those of a previous study by Dietz et al. [21]. Using the symphysis pubis as a reference, Dietz et al. reported that tape position varied from 30 mm above to 12.7 mm below the symphysis at rest and from 15 mm above to 18.7 mm below the symphysis on Valsalva [21]. Recurrent SUI was weakly associated with the horizontal distance of the tape from the symphysis pubis (*p* = 0.048), whereas more cranial tapes were weakly associated with urge incontinence (*p* = 0.03) 21]. Yang et al. also used the symphysis pubis as a reference and found that, in patients with successful outcomes of TOT, the tape position became more caudal relative to the symphysis pubis at Valsalva, but still remained at the middle portion of the urethra [22,23], similar to the findings in our study.

In a retrospective study Huang et al. [24] showed, instead, that the resting bladder neck location did not change significantly 5 years after TOT; also upon coughing, it was similar perioperatively.

We believe that the reference for the correct tape position is the urethra, as the continence mechanism is determined by the interaction of the urethra with the sling.

Mid-urethral positioning of the sling allows the tape to act as a fulcrum, either kinking the urethra [24, 25] or enhancing the increase in intraurethral pressure with stress [26, 27]. One study reported dynamic kinking of the urethra with stress in 87-92% of women who underwent MUS implantation [26].

The concordance of urethral movement with the sling is a factor for ensuring surgical success. In our study, the urethra moved concordantly relative to the sling in all continent women, whereas in the incontinent group, as well as in the incontinent women studied by Hegde et al. [11], urethral movement was discordant relative to the sling. The concordance of urethral movement with the sling indicates how effectively the sling is fixed to the mid-urethral soft tissue. This is another essential mechanism for dynamic urethral compression during increased intra-abdominal pressure. TOT failure may be associated with a sling that is not adequately attached to the urethral tissue. In these cases, the urethra and surrounding tissue move independently of the sling at Valsalva [20] and the urethrovesical junction moves distal to the sling during stress, as shown in our data. If the urethra moves concordantly with the sling, but the sling is positioned proximal to the bladder neck, patients could experience voiding symptoms. In our study, women with de novo voiding symptoms had a proximal sling. In this case, the immobilization of the bladder neck could simulate a Burch colposuspension, creating a highly unphysiological appearance [28].

In patients with SUI, funneling of the internal urethral meatus may be observed at Valsalva and is associated with poor urethral closure pressure [29, 30]. This may explain why funneling was present more in patients in whom surgical treatments have failed. However, bladder neck funneling was also present in continent patients, suggesting that multiple factors might be needed to make a sling implant fail (asymmetric arm, distal position, open bladder neck, etc.)  [31].

Our results should be interpreted with the acknowledgement that our study is limited by a small sample size, and that the analysis is based on objective, but not subjective SUI. The strengths of our study are its prospective design, long-term analysis, correlation with functional outcomes, and study of the TOT sling, which make it unique among other works in the literature.

## Conclusion

A correct TOT sling position along the urethra and the concordant movement of the urethra with the sling seem to play an important role in the long-term outcomes of MUS implantation as a treatment for SUI. These characteristics, as assessed by transperineal ultrasound, do not change in the 6 years post-surgery, and a correct surgical technique is therefore mandatory for obtaining the best results.
